# Metformin inhibits high glucose‐induced apoptosis of renal podocyte through regulating miR‐34a/SIRT1 axis

**DOI:** 10.1002/iid3.1053

**Published:** 2024-01-19

**Authors:** Xudong Zhuang, Zhuye Sun, Huasheng Du, Tianhui Zhou, Jing Zou, Wei Fu

**Affiliations:** ^1^ Department of Dialysis Linyi Traditional Chinese Medicine Hospital Linyi Shandong China; ^2^ Department of Pharmacy Rizhao Hospital of Traditional Chinese Medicine Rizhao Shandong China; ^3^ Department of Nephrology Qingdao Municipal Hospital Qingdao Shandong China; ^4^ Beijing University of Chinese Medicine Beijing China; ^5^ Department of Drug Dispensing Zibo Central Hospital Zibo Shandong China

**Keywords:** DN, metformin, miR‐34a, podocyte, SIRT1

## Abstract

**Background:**

Previous studies have reported SIRT1 was inversely modulated by miR‐34a, However, mechanism of metformin (MFN)'s renal podocyte protection under high glucose (HG) conditions and the connection between miR‐34a and SIRT1 expression in diabetic nephropathy (DN) remain unclear.

**Method:**

We aimed to further elucidate the role of miR‐34a in HG‐treated podocytes in DN. A conditionally immortalized human podocyte cell line was cultivated in d‐glucose (30 mM).

**Results:**

Microarray and RT‐qPCR revealed that miR‐34a was downregulated in HG‐treated podocytes. Additionally, miR‐34a levels increased in MFN‐treated HG‐induced podocytes. CCK‐8 assay, colony formation assay, flow cytometry, and Western blot detection showed that HG treatment reduced cell viability and promoted via HG treatment, and MFN treatment reversed this phenotypic change. MiR‐34a upregulation caused restored cell viability and suppressed cell apoptosis in HG‐treated podocytes, and miR‐34a downregulation led to damaged cell survival and induced apoptosis in MFN‐administered and HG‐treated podocytes. The dual luciferase reporter assay showed that SIRT1 3′‐UTR was a direct miR‐34a target. Further studies demonstrated an elevation in SIRT1 levels in HG‐exposed podocytes, whereas MFN treatment decreased SIRT1 levels. In addition, miR‐34a upregulation led to reduced SIRT1 expression, whereas miR‐34a inhibition increased SIRT1 levels in cells. MFN‐induced miR‐34a suppresses podocyte apoptosis under HG conditions by acting on SIRT1.

**Conclusion:**

This study proposes a promising approach to interpret the mechanisms of action of the MFN‐miR‐34a axis involved in DN.

## Graphical abstract

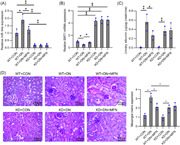

## 1 INTRODUCTION

Diabetic nephropathy (DN) is a major debilitating complication of type 1 and 2 diabetes that can develop into terminal renal disease.^1^ Persistent microalbuminuria is extensively used as an early DN biomarker, which indicates a gradual decrease in renal function.^2^ Podocytes are terminally differentiated cells that cannot proliferate^3^ and are an obstacle to glomerular filtration, along with the glomerular basement membrane and endothelial cells.^4^ Some studies have suggested that podocyte apoptosis is associated with the reduced expression of nephrin, podocin, and slit‐relevant proteins,^5^ leading to massive proteinuria in DN.^6^ Thus, podocyte‐based treatments that prevent DN in the early stages require further research.

Metformin (MFN), a first‐tier hypoglycemic drug for type 2 diabetes (T2D) treatment, can prevent and postpone DN occurrence and progression via several mechanisms, including elevated insulin resistance and glucolipid metabolism, restoration of renal tissue podocalyxin, and several other non‐hypoglycemic mechanisms.^7^, ^8^ In addition, MFN has been explored for the treatment of other diseases.^9^, ^10^ MFN can suppress renal fibrosis^11^ and decrease microalbuminuria in T2DM patients.^12^

miRNAs are short (∼22 nucleotides) noncoding RNAs that usually connect with 39 untranslated regions of mRNAs, functioning mostly at the posttranscriptional level and suppressing mRNA translation and stability.^13^ miRNAs are closely related to diabetes pathology and renal impairment.^14^, ^15^, ^16^ Microarray findings have shown that miR‐34a expression is increased in DN.^17^ In several other studies, miR‐34a was closely associated with cell proliferation.^17^, ^18^ Zhang et al. showed that miR‐34a might influence the proliferation of mesangial cells in DN.^19^ Many studies have demonstrated the regulatory role of MFN in miR‐34a in various cases. Do et al. indicated that MFN could elicit miR‐34a to reduce the SIRT1/Pgc‐1α/Nrf2 pathway, elevating wild‐type (WT) p53 cancer cells' susceptibility.^20^ However, another study indicated that MFN inhibits miR‐34a expression to regulate Egr1 in high glucose (HG)‐elicited rat mesangial cells.^21^ Additionally, SIRT1, which protects podocytes from injury during DN,^22^ has been reported to be inversely modulated by miR‐34a.^22^, ^23^, ^24^ However, the MFN‐miR‐34a axis function and mechanism of renal podocyte protection under HG conditions and the connection between miR‐34a and SIRT1 expression in DN remain unclear.

Based on these findings, the aim of this study was to clarify the effect of the MFN‐miR‐34a axis on podocyte survival and apoptosis under HG‐cultivated conditions in vitro and probe the influence of this axis on diabetes‐induced glomerular impairment in vivo.

## 2 MATERIALS AND METHODS

### 2.1 Human podocyte cell line

Human podocytes were cultivated as described previously,^25^ collected 1 day after transfection, and processed for total protein and RNA isolation, cell viability, and apoptosis.

Podocytes were cultivated in RPMI 1640 medium, and 10% FBS was added in a damp condition with 5% CO_2_ at 33°C. After cultivation to an 80%–90% confluence, the podocytes were subcultured for 10–14 days under similar conditions to induce cell differentiation. After starvation with serum for 0.5 day, cells were subjected to normal glucose (5 mmol/L d‐glucose, NG) or the indicated HG (30 mmol/L d‐glucose) for 3 days.

### 2.2 miRNA microarray

Total RNA was separated using TRIzol reagent, and RNA quality was evaluated via capillary electrophoresis. Small RNA sequencing libraries were obtained using the Small RNA Library Prep Set for Illumina as the relevant guide and quantified using the Agilent Bioanalyzer 2100 system. The quality of the original sequence data was analyzed. To eliminate low‐quality results, we unloaded adapters by using Cutadapt 1.2.1, and the corresponding sequences were trimmed. The clean reads obtained were sifted as miRNA at 21–22 nt and located to the reference sequence via Bowtie 2. The roles of new miRNAs were investigated using miRDeep2 2.0.0.8. Sequences with differential expressions were utilized to calculate differential expressions and assess the data significance of the changes between the case and control specimens.

### 2.3 Cell transfection

Hsa‐miR‐34a mimic and inhibitor and their relative controls were chemically synthesized by GenePharma. Cells were transfected with all plasmids and oligonucleotides by using Lipofectamine 2000 according to the manufacturer's instructions.

### 2.4 **Reverse transcription‐quantitative polymerase chain reaction** (RT‐qPCR)

Total RNA, such as microRNA, was isolated from cultivated tissues and cells by using TRIzol reagent. miRNA or mRNA RT‐qPCR was conducted using One‐Step TB Green PrimeScript RT‐PCR Kit (Takara) or SYBR Premix ExTaqTM and an Applied Biosystems 7500 Fast real‐time PCR system. The PCR cycle procedures were as follows: 10 min at 95°C, 40 cycles of 15 s at 95°C, and 35 s at 60°C. GAPDH and U6 snRNAs served as endogenous normalization references. Each specimen was tested in triplicate, and all experiments were repeated at less than three times. Relative gene expression was determined using the comparative CT method.

### 2.5 Western blot (WB)

Cells were collected and lysed in RIPA buffer containing PMSF protease inhibitors. The BCA assay (Beyotime Institute of Biotechnology) measured the protein concentrations as per relevant guidance. Specimens were boiled in 5× sample buffer for 5 min at 100°C. The same amounts of protein (50 μg per sample) were isolated by SDS‐PAGE (8%–12%) and subsequently added to PVDF membranes at a 200 mA constant current. Blots were blocked using 5% BSA in Tris‐buffered saline‐Tween20 (pH7.6) for 120 min at RT. Next, membranes were cultivated with primary antibodies at 4°C overnight and exposed to anti‐rabbit/mouse (1:10,000) secondary antibodies at RT for 60 min. Immunoreactive bands were observed using a bio‐imaging system, and the relative gray value was analyzed using Gelpro32.

### 2.6 Cell proliferation and colony formation assay (CFA)

Cell viability was assessed using CCK‐8 assay as the relevant guidance. CCK‐8 (10 μL) was added after the cells were inoculated into 96‐well plates. Next, the cells were cultured for another 120 min at 37°C. The OD_450 nm_ (optical density) was determined using an infinite M200 (Tecan).

Cell viability was evaluated using CFA. After culturing for 7 days in six‐well plates, the cells were treated with formaldehyde (4%) fixation for 20 min and subjected to crystal violet (1.0%) staining.

### 2.7 Analysis of apoptosis by flow cytometry (FC)

Podocyte apoptosis was assessed 2 days after exposure to the various treatments. In brief, podocytes were trypsinized and centrifuged at RT (1000 rpm for 5 min). Subsequently, 1× binding buffer was added to the precipitate, followed by the Annexin V FITC stock solution. Cells were cultivated for 10 min at 4°C. Propidium iodide (PI) was added immediately. A total of 20,000 cells per specimen were used for FC, and the data obtained were used to measure the proportion of apoptotic cells.

### 2.8 Dual luciferase reporter assay (DLRA)

A firefly luciferase reporter assay was used to confirm whether miR‐34a directly targets the SIRT1 3′‐UTR through the pGL3 control vector. Initially, the WT 3′‐UTR sequence of SIRT1 with putative miR‐34a target sites or mutated 3′‐UTR established by substituting several miR‐34a binding sites with other nucleotides was prepared and subcloned into the pGL3 control vector XbaI and FseI sites. For the reporter assay, pGL3 vectors (0.1 mg) with WT or mutated SIRT1 3′‐UTR were cotransfected with miR‐34a or NC mimic into HEK293 cells. After transfection for 2 days, the cells were collected, and luciferase activity was determined using the DLRA System (Promega).

### 2.9 Animals

Every procedure was approved by the Animal Care and Use Committee of Zibo Central Hospital. C57BL/6J male mice were provided by Silaike and were injected with STZ to induce diabetes. In brief, after fasting for 5 h, the mice were administered STZ (55 mg/kg, I.P) for 5 days. Blood glucose levels were examined 7 days after the last STZ administration. Mice with a nonfasting blood glucose level >400 mg/dL were considered diabetic.

After dissolution in saline (5 mg/mL), LNA‐anti‐miR‐34a and the control were injected into the tail vein (2 mg/kg/mouse). Mice were injected once per week for 24 weeks after the occurrence of diabetes.

The mice were then randomly divided into six groups. Each group comprised six mice: (1) control mice without diabetes, (2) STZ mice exposed to vehicle, (3) STZ mice exposed to MFN, (4) control mice without diabetes, (5) STZ mice with LNA‐anti‐miR‐34a injection exposed to vehicle, and (6) STZ control mice with LNA‐anti‐miR‐34a exposed to MFN. MFN (350 mg/kg body weight) was administered orally once daily for 24 weeks. For each experiment, body weight and glycemia in the diabetic mice were measured twice per week. Mice were housed separately in metabolic cages, and urine specimens were collected 1 day later. The mice were killed 24 weeks after STZ administration, and kidney tissues and blood serum were collected for the next study.

### 2.10 Kidney histology

Kidney specimens were treated with formalin (10%) fixation and embedded in paraffin. Sections with periodic acid‐Schiff staining were examined to measure the mesangial area and glomerular volume.

### 2.11 Urine albumin analysis

Urine albumin and creatinine levels were determined using an ELISA kit (Bethyl Laboratory Inc.) and a colorimetric assay kit (Bioassays Systems), respectively. The excretion rate of urine albumin is expressed as the albumin/creatinine ratio.

### 2.12 Data analysis

All quantitative data are displayed as mean ± SD. A two‐tailed Student's *t* test or one‐way analysis of variance (ANOVA) was conducted to analyze the data by using SPSS 16.0. Statistical significance was set at *p* < .05.

## 3 RESULTS

### 3.1 miR‐34a expression was reduced in MFN‐administrated podocytes with HG treatment

**Figure 1:**
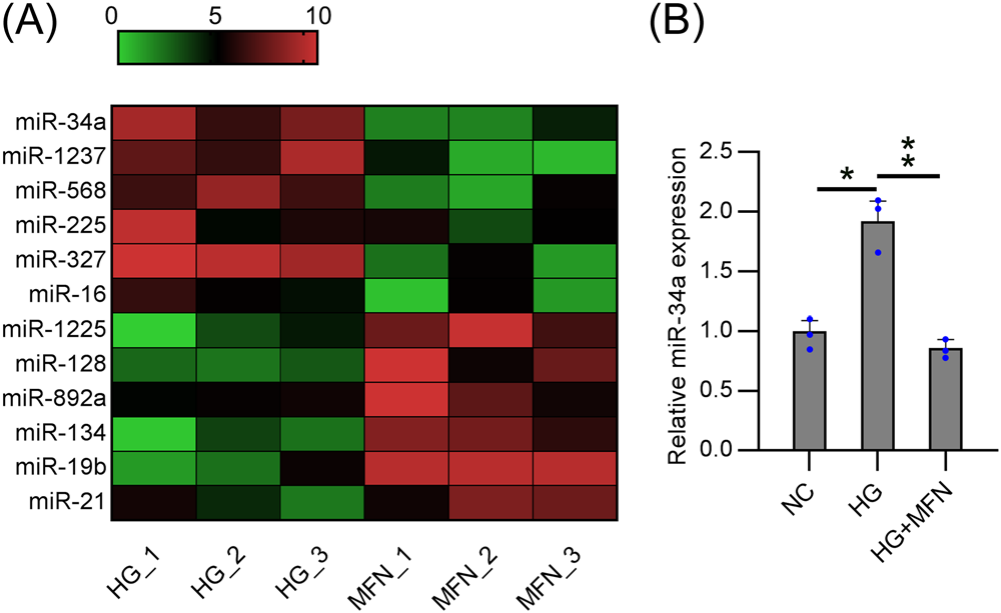
Dysregulated miR‐34a expression in HG‐treated podocytes with MFN administration. (A) miRNA microarray of differentially regulated miRNAs in HG‐treated podocytes w/wo MFN treatment. (B) RT‐qPCR was utilized for determining the miR‐34a levels in HG‐treated podocytes w/wo MFN treatment. Data were obtained from the results of three separate experiments and displayed as mean ± SD. ANOVA test was applied to compare the differences among multiple groups. *p < .05, **p < .01, relative to the indicated group. HG, high glucose; MFN, metformin.

miRNA microarray analysis showed the top 6 upregulated and downregulated miRs between HG‐treated podocytes and MFN‐administered HG‐treated podocytes. The data revealed that miR‐34a expression was reduced after MFN administration single HG‐treated cells (Figure 1A). Furthermore, RT‐qPCR confirmed that miR‐34a expression in HG‐treated podocytes was upregulated relative to that in untreated podocytes. MFN treatment significantly downregulated miR‐34a levels in cells. Our data showed that HG‐induced miR‐34a expression decreased after MFN exposure (Figure 1B).

### 3.2 Effects of miR‐34a on cell viability and death of MFN‐administrated and HG‐treated podocytes

**Figure 2:**
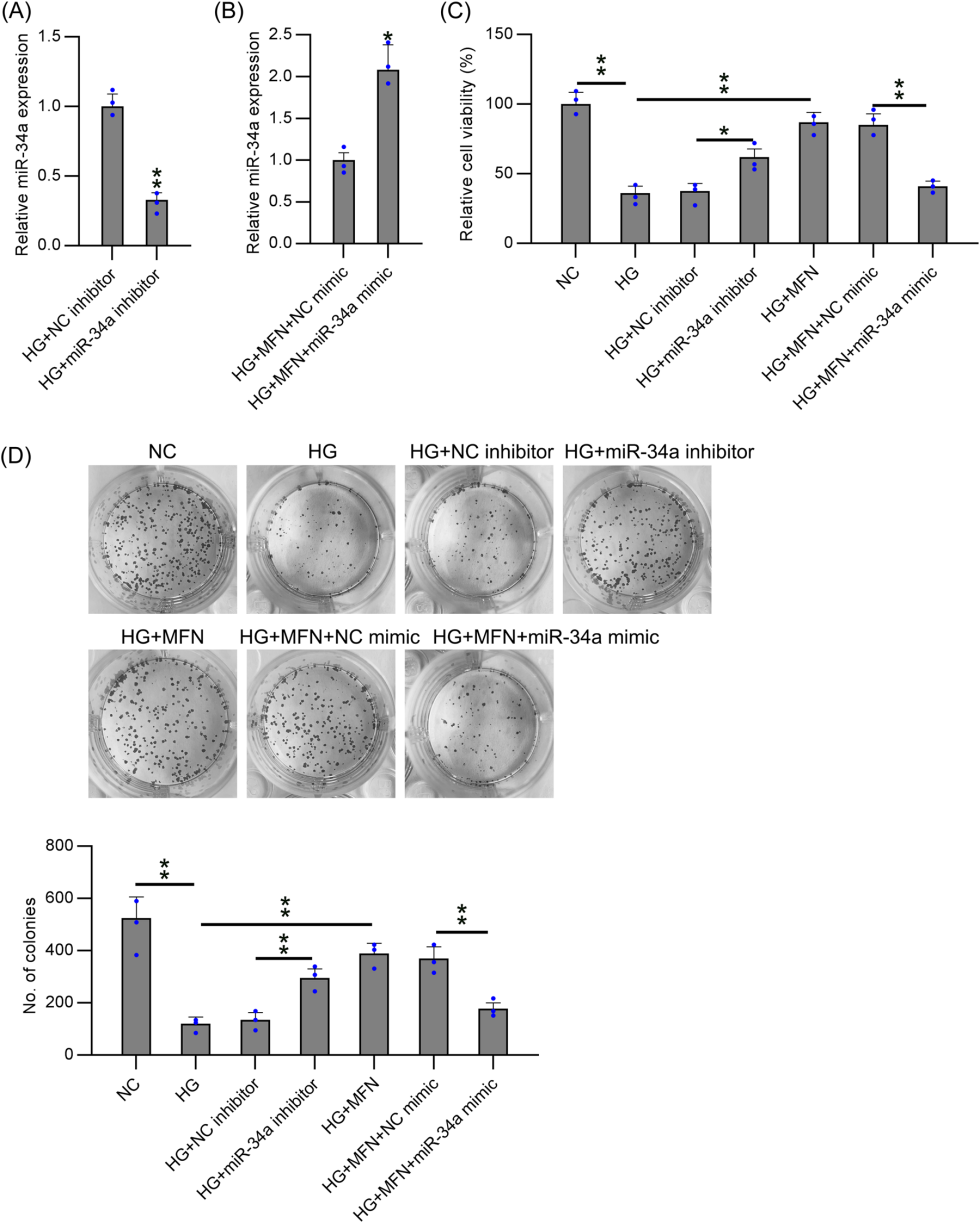
Up‐ or downregulation of miR‐34a and its effect on cell viability in podocytes with various treatments. HG‐treated podocytes were subjected to miR‐34a or NC inhibitor transfection for 1 day. MFN‐administrated and HG‐treated podocytes were subjected to miR‐34a or NC mimic transfection for 1 day. (A, B) RT‐qPCR was utilized for determining the miR‐34a level in podocytes with various treatments. (C) CCK‐8 assay showed the impact of different transfections and treatments on the cell viability of podocytes. (D) CFA was used to determine the growth rate of the podocytes with various treatments. Data were obtained from the results of three separate experiments and displayed as mean ± SD. Students's *t* test or ANOVA was applied to compare the difference between two groups or multiple groups, respectively. *p < .05, **p < .01, relative to the indicated group. CFA, colony formation assay; HG, high glucose; MFN, metformin.

To investigate the influence of miR‐34a on HG‐induced podocyte proliferation and viability, we downregulated miR‐34a expression in HG‐treated cells and upregulated miR‐34a in MFN‐administered and HG‐treated podocytes to establish a loss‐ and gain‐of‐function study. These dysregulations were confirmed using real‐time PCR (Figure 2A,B).

The effect of miR‐34a on the viability of HG‐treated podocytes without MFN administration was assessed using the CCK‐8 assay. Relative to the NC inhibitor transfection group, miR‐34a inhibition dramatically restored cell viability, which was impaired by HG treatment (Figure 2C). Compared with the NC mimic group, upregulation of miR‐34a led to attenuated cell viability, counteracting the protective role of MFN on the viability of HG‐treated podocytes (Figure 2C). CFA was used to measure the growth rate of podocytes after various treatments. The results showed a trend similar to that of the CCK‐8 assay (Figure 2D).

**Figure 3:**
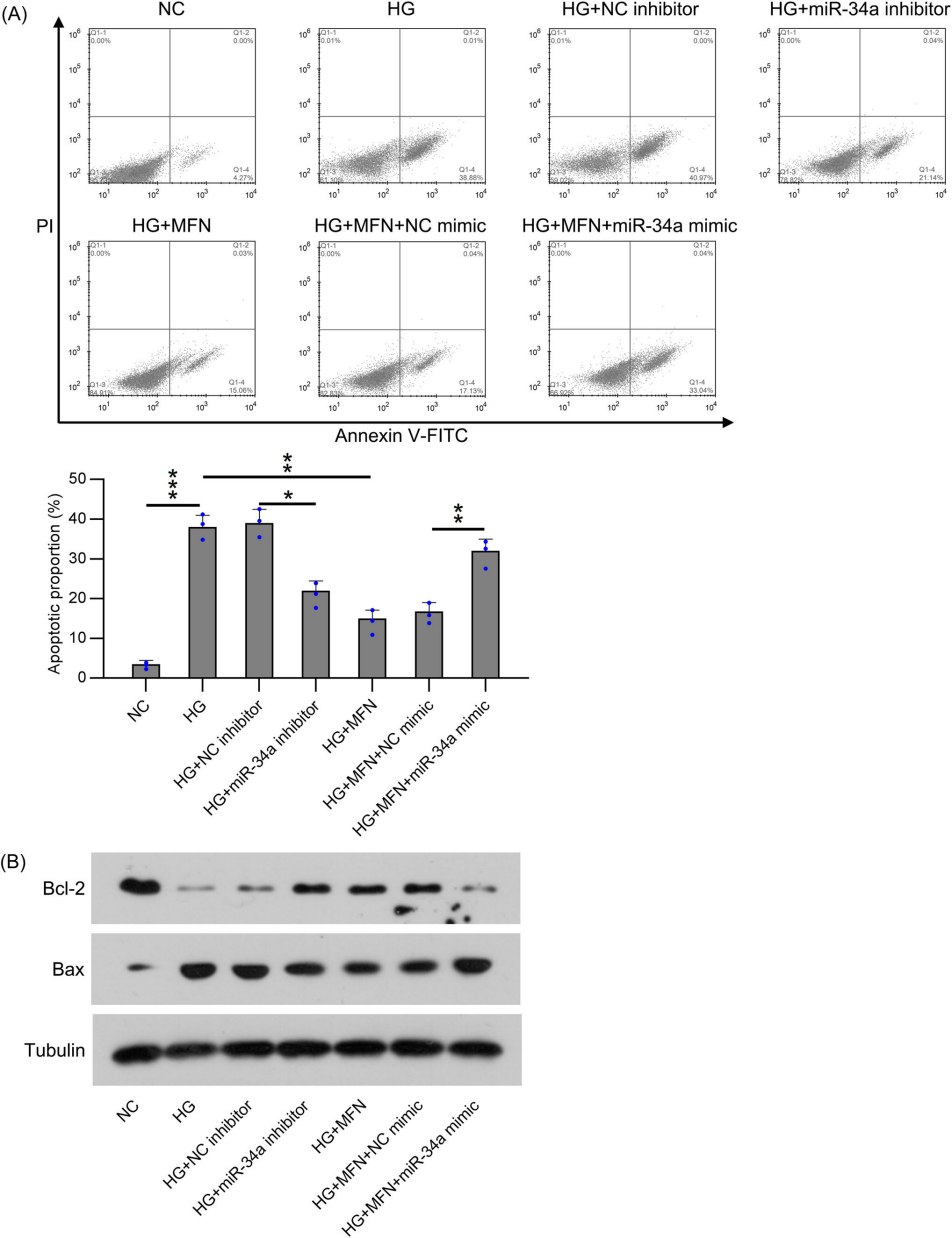
miR‐34a regulates cell apoptosis in MFN‐administrated and HG‐treated podocytes. (A) FC analysis was used to show the apoptosis proportion in podocytes. (B) WB analysis showed the expression apoptotic related Bcl2 and Bax protein in podocytes. Data were obtained from the results of three separate experiments and displayed as mean ± SD. ANOVA test was applied to compare the difference among multiple groups. *p < .05, **p < .01, ***p < .001, relative to the indicated group. FC, flow cytometry; HG, high glucose; MFN, metformin; WB, Western blot.

Next, on the basis of the aforementioned observations, we detected the apoptotic proportion of podocytes by using Annexin V‐FITC and PI FC. We found that the inhibition of miR‐34a reduced the apoptotic proportion of podocytes after HG treatment, compared with the NC inhibitor transfection groups (Figure 3A). Additionally, upregulation of miR‐34a significantly recovered the percentage of apoptotic cells in HG podocytes after MFN administration (Figure 3A). We also detected the expression of apoptosis‐related proteins (Bcl2 and Bax) by using WB blotting. Our data showed that Bcl2 was obviously reduced, Bax was increased upon HG stimulation, and MFN administration reversed this phenotypic change. miR‐34a inhibition significantly promoted Bcl2 and reduced Bax in HG‐stimulated podocytes, whereas miR‐34a upregulation inhibited Bcl2 and elevated Bax levels in MFN‐treated HG cells (Figure 3B). These findings indicate that miR‐34a may induce apoptosis in HG‐treated podocytes.

### 3.3 MiR‐34a directly targets SIRT1 3′‐UTR

**Figure 4:**
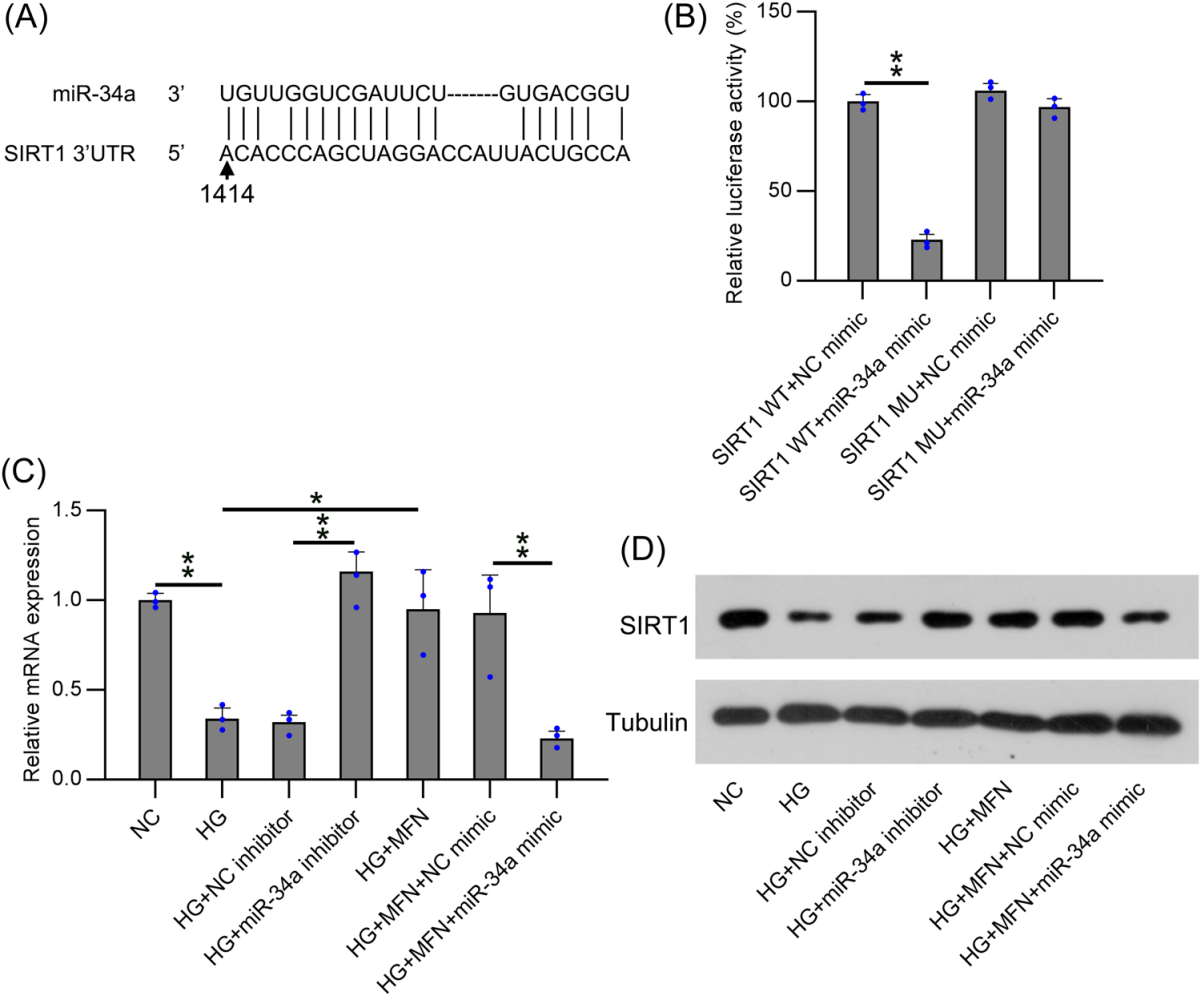
miR‐34a targets SIRT1 3′‐UTR. (A) Bioinformatic analysis indicated that miR‐34a had a binding site in SIRT1 3′‐UTR. (B) DLRA was conducted after HEK293T cells were cotransfected with a luciferase reporter containing either a mutant from or WT SIRT1, and a miR‐34a mimic. Real‐time PCR (C) and WB (D) analyses showed the SIRT1 level in podocytes with indicated treatments. Data were obtained from the results of three separate experiments and displayed as mean ± SD. ANOVA test was applied to compare the difference among multiple groups. *p < .05, **p < .01, relative to the indicated group. DLRA; dual luciferase reporter assay; HG, high glucose; MFN, metformin; WB, Western blot; WT, wild‐type.

The accumulating evidence suggests that miR‐34a can regulate the target SIRT1 3′‐UTR.^22^, ^26^, ^27^, ^28^, ^29^ Our bioinformatic prediction indicated that miR‐34a might target the SIRT1 3′‐UTR (Figure 4A). The direct correlation between SIRT1 and miR‐34a was further validated using DLRA (Figure 4B). The luciferase activity was repressed by approximately 75% in cells subjected to WT 3′‐UTR SIRT1‐fused miR‐34a mimic transfection as relative to that in cells from the control groups.

Furthermore, RT‐qPCR and WB were conducted to detect SIRT1 expression in podocytes with different treatments. We found that SIRT1 expression levels were reduced in the HG‐treated group, whereas MFN administration recovered SIRT1 in HG‐induced podocytes. Similarly, the miR‐34a inhibitor transfection remarkably promoted SIRT1 expression in HG cells, and the miR‐34a mimic reduced SIRT1 in MFN‐administered and HG‐induced podocytes (Figure 4C,D). These observations indicate that the SIRT1 3′‐UTR is an miR‐34a target. HG treatment of podocytes caused a significant reduction in SIRT1 expression, and MFN administration promoted SIRT1 expression in podocytes.

### 3.4 MiR‐34a knockdown abolished MFN‐exert protective function in diabetes‐induced glomerulus impairment

**Figure 5:**
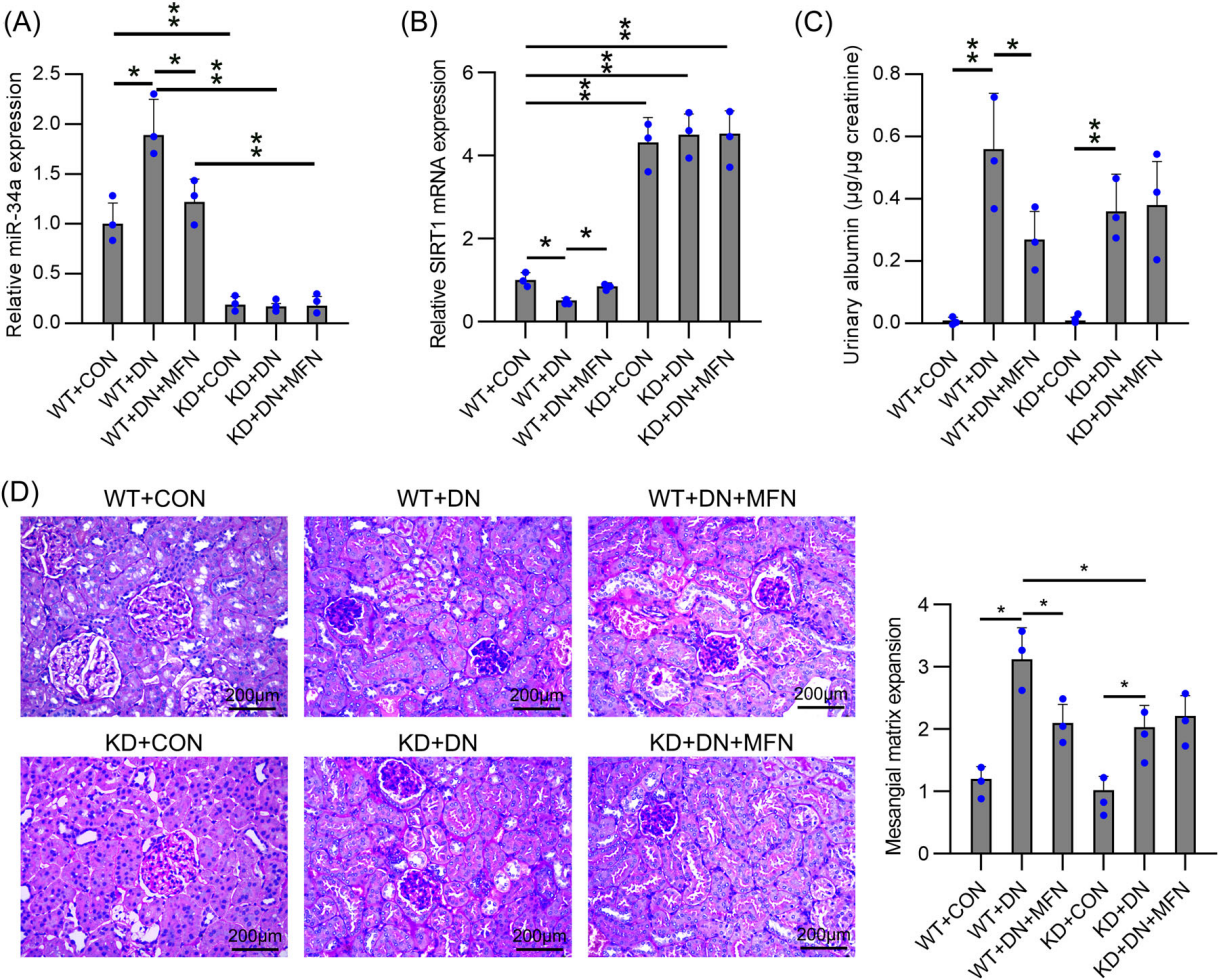
MiR‐34a knockdown can prevent diabetes‐elicited glomerulus impairment in vivo and abolished the protective function of MFN. (A) miR‐34a knockdown by LNA‐modified miR‐34a inhibitor demonstrated through RT‐qPCR. (B) Expression of SIRT1 mRNA in kidney tissue. (C) Urine albumin/creatinine values from nondiabetic and diabetic mice w/wo miR‐34a inhibition or MFN administration at 24 weeks post STZ injection. (D) Representative micrographs of kidney sections stained with PAS and glomerular volume and mesangial matrix expansion quantification from arbitrarily chosen glomeruli in the indicated groups. Data were obtained from the results of three separate experiments and displayed as mean ± SD. ANOVA test was applied to compare the difference among multiple groups. *p < .05, **p < .01, relative to the indicated group. MFN, metformin.

Next, we confirmed the function of miR‐34a in glomeruli impairment in the DN model: NC or miR‐34a inhibitor modified by locked nucleic acid (LNA) was administered via the tail vein every week for 24 weeks after continuous STZ administration (I.P, 55 mg/kg). MFN (350 mg/kg) was administered orally once daily for 24 weeks. STZ mice developed DN at 24 weeks with upregulated miR‐34a expression in glomeruli, whereas oral administration of MFN reduced miR‐34a levels and upregulated SIRT1 mRNA (Figure 5A,B). In miR‐34a knockdown mice, miR‐34a levels in the glomerulus were maintained at a low level while SIRT1 mRNA maintained at a high level throughout the experimental period (Figure 5A,B). The albumin‐to‐creatinine ratio (ACR) examined showed that DN modeling increased the ACR value, which could be reduced by MFN administration. Furthermore, the ACR value in miR‐34a knockdown mice was reduced upon DN modeling relative to that in WT mice. However, no difference was observed between DN and DN + MFN in ACR levels in miR‐34a knockdown mice (Figure 5C). DN glomerular mesangial hypertrophy and mesangial expansion in the DN + MFN group was lower than that in the DN group in WT mice at the 24th week. MiR‐34a knockdown also ameliorated DN‐induced hypertrophy and mesangial expansion, and MFN showed no significant improvement in glomerular mesangial hypertrophy and mesangial expansion in miR‐34a knockdown mice (Figure 5D).

## 4 DISCUSSION

DN is a severe complication of type 1 and type 2 diabetes mellitus^30^ and a commonly observed trigger of terminal renal disease. MFN, together with lifestyle changes, is regarded as the first‐tier oral treatment in the current guidelines of the European Association of the Study of Diabetes and American Diabetes Association.^31^, ^32^ The major effect of this medicine is a sharp reduction in hepatic glucose generation, largely via mild and temporary suppression of the mitochondrial respiratory chain complex I.^33^ Studies have suggested that MFN exhibits a specific protective effect against DN, independent of its hypoglycemic effect.^34^, ^35^ Accumulating evidence indicates that MFN can shield kidney tubular cells and podocytes from injury and prevent DN progression.^36^ However, the ability of MFN to prevent clinical DKD requires further investigation. This study indicated that HG stimulation elicits apoptosis in podocytes, suppressing cell growth and viability. Administration of MFN significantly reduced apoptosis and restored cell viability in HG‐treated podocytes, suggesting a protective role for MFN in HG‐treated podocytes.

A study demonstrated that miR‐34a/SIRT1 axis suppression ameliorates podocyte injury in DN.^22^ By contrast, in WT p53 cancer and HG‐treated rat mesangial cells, the regulatory effect of MFN on miR‐34a expression was the opposite. In p53 cancer cells, MFN treatment caused miR‐34a upregulation,^20^ whereas miR‐34a expression was reduced in MFN.^21^ Therefore, we hypothesized that MFN exerts its function in podocyte protection by regulating miR‐34a expression. Zhang et al. was indicated to negatively regulate the expression of lactate dehydrogenase A, which consequently inhibited lactate dehydrogenase A‑dependent glucose uptake in the cancer cells,^37^ suggesting that miR‑34a inhibits liver cancer cell growth by reprogramming glucose metabolism, and owning the capacity to regulating glucose metabolism. In this study, we observed that miR‐34a expression in podocytes was elevated after HG stimulation, but this elevation was partially reversed by MFN administration, similar to that reported in HG‐treated mesangial cells.^21^ Restoration of miR‐34a expression in MFN‐administrated podocytes counteracted the impact of MFN on cell viability and apoptosis. Additionally, in vivo animal experiments indicated that glomerular mesangial hypertrophy caused by DN modeling could be ameliorated by miR‐34a knockdown, which abolished the effect of MFN on hypertrophy. These data indicate that MFN plays a role in podocyte protection in miR‐34a dependent manner.

Our experimental results show that HG stimulation upregulated miR‐34a and suppressed cell viability by upregulating apoptosis in podocytes. MFN attenuates HG‐stimulated cell death in podocytes by downregulating miR‐34a. Additionally, our data showed that SIRT1 expression was positively correlated with MFN treatment and inversely correlated with miR‐34a expression. One of limitations of our study is lacking the evidence and mechanism regarding SIRT1 in the protective role of MFN in HG‐induced podocyte injury was not explored in this study; thus, we plan to investigate this topic in our future study.

## AUTHOR CONTRIBUTIONS

**Xudong Zhuang**: Conceptualization; data curation; formal analysis; writing—original draft; writing—review and editing. **Zhuye Sun**: Conceptualization; data curation; formal analysis; writing—original draft; writing—review and editing. **Huasheng Du**: Formal analysis; investigation. **Tianhui Zhou**: Formal analysis; methodology. **Jing Zou**: Formal analysis; methodology. **Wei Fu**: Conceptualization; data curation; formal analysis; methodology.

## CONFLICT OF INTEREST STATEMENT

The authors declare no conflict of interest.

## ETHICS STATEMENT

Every procedure was approved by the Animal Care and Use Committee of Zibo Central Hospital.

## Data Availability

All data generated or used during the study appear in the submitted article.
